# Effect of temperature on wood modification with citric acid

**DOI:** 10.1515/hf-2025-0127

**Published:** 2026-01-19

**Authors:** Assira Keralta, Johannes Karthäuser, Jérémy Winninger, Julien Chamberland, Yu Ogawa, Yoshiharu Nishiyama, Marie-Josée Dumont, Véronic Landry, Holger Militz

**Affiliations:** Renewable Materials Research Center, Department of Wood and Forest Sciences, 4440 Université Laval, 2425 Rue de la Terrasse, Quebec City, QC, G1V 0A6, Canada; Wood Biology and Wood Products, University of Göttingen, Büsgenweg 4, 37077 Göttingen, Germany; STELA Dairy Research Center, Institute of Nutrition and Functional Foods (INAF), Department of Food Sciences, 4440 Université Laval, 2425 Rue de l’Agriculture, Quebec City, QC, G1V 0A6, Canada; Department of Sustainable and Bio-inspired Materials, Max Planck Institute of Colloids and Interfaces, Am Mühlenberg 1, 14476 Potsdam, Germany; CERMAV, CNRS, Université Grenoble Alpes, Grenoble, 38000, France; Chemical Engineering Department, 4440 Université Laval, 1065 Avenue de la Médecine, Quebec City, QC, G1V 0A6, Canada

**Keywords:** wood modification, citric acid, curing temperature, esterification, crosslinking

## Abstract

Citric acid (CA) has emerged as a promising biosourced reagent for wood modification, improving the wood’s dimensional stability and durability. However, CA treatments are often associated with reduced mechanical strength, and the specific role of curing temperature in this balance remains insufficiently explored. This study investigates the effect of curing temperature on the chemical, physical, and mechanical behavior of CA-modified wood. Scots pine (*Pinus sylvestris* L.) sapwood samples were impregnated with 30 % CA solution and cured at 100, 120, 140, and 160 °C for 24 h. Chemical changes were analyzed by FTIR and solid-state ^13^C NMR, while physical and mechanical performance was assessed through weight gain, cell wall bulking, anti-swelling efficiency, moisture exclusion efficiency, Brinell hardness, and three-point bending strength. Results demonstrated that esterification between CA and wood hydroxy groups increased with temperature, enhancing dimensional stability, especially at 140–160 °C. However, curing at 160 °C also promoted acid-catalyzed hydrolysis of cell wall components, leading to significant reductions in bending strength. The best compromise between stability and strength was achieved at 140 °C. These findings highlight curing temperature as a critical factor for optimizing CA treatments, providing a pathway for industrially viable wood modification strategies that balance performance with sustainability.

## 1 Introduction

Citric acid (CA) is widely known for its applications in the food, pharmaceutical, and cosmetic industries, owing to its non-toxicity and biodegradability (Ciriminna et al. 2017). CA, a naturally occurring organic acid, is primarily produced through fermentation processes of carbohydrate-rich substrates (such as molasses, starch hydrolysates, or glucose) using *Aspergillus niger* (Lee et al. 2020). Recently, CA has attracted interest in the field of wood modification due to its potential as a sustainable, environmentally friendly alternative to conventional wood treatments, which often rely on synthetic chemicals or toxic substances (Cahyono and Syahidah 2019).

The use of CA in wood modification leverages its ability to form ester bonds with the hydroxy groups in cellulose in the wood matrix. This process can improve the wood’s dimensional stability, and resistance to moisture (Augustina et al. 2023; Cruz-Lopes et al. 2025; L’Hostis et al. 2018). However, the modification leads to a loss of wood mechanical properties, especially the bending strength (Augustina et al. 2023; Dong et al. 2023; Feng et al. 2014; L’Hostis et al. 2018), limiting its use as modification treatment. The loss in mechanical properties during the modification with CA was explained by the crosslinking between CA and OH group in the wood matrix, which decreases wood flexibility and increases its brittleness (Cruz-Lopes et al. 2025). It is why, when CA is combined with polyols such as sorbitol or glycerol, the crosslinking density between CA and wood-free OH groups decreases due to its reaction with these polyols. This reduction in crosslinking density contributes to improved flexibility and helps mitigate brittleness as observed by Hötte and Militz (2024). Potential hydrolysis of wood components at these conditions can also explain the loss in wood mechanical properties. Two key parameters are essential during this reaction: CA concentration and curing temperature.

**Figure 1:**
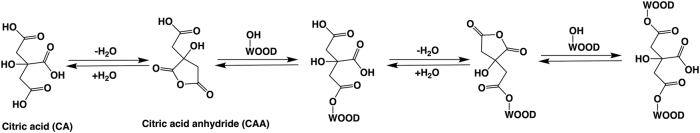
CA reaction pathway with wood according to (Bischof Vukusic et al. 2006).

The esterification reaction pathway between CA and wood is well-known (Figure 1). It involves a two-step esterification: an anhydride is first formed, followed by its reaction with the free hydroxy groups of the wood matrix (Bischof Vukusic et al. 2006; Schramm and Rinderer 1999). In the absence of any catalyst, a temperature around the CA melting point (156 °C) is generally required to trigger the reaction (Lee et al. 2020). As CA is slightly more acidic than typical carboxylic acids (Apelblat 2014), potential hydrolysis of wood components may occur during curing. CA can also auto-polymerize to form CA ester when heated (Tsioptsias et al. 2024).

During wood modification with CA solely catalyzed by heat, higher temperatures led to a higher degree of CA reaction with wood, which reduces wood hygroscopicity and hence, enhances its dimensional stability and durability. However, higher temperatures could also enhance the hydrolysis of wood components, leading to a decrease in wood mechanical properties. Therefore, this study aims to investigate the effect of temperature on CA wood modification to determine the most suitable treatment temperature for effective wood modification.

## 2 Materials and methods

### 2.1 Materials

CA monohydrate (approx. 97 % purity) was obtained from PanReac AppliChem (Darmstadt, Germany) and used as received. Scots pine (*Pinus sylvestris* L.) sapwood samples were prepared and cut according to the specifications in Table 1.

**Table 1:** Wood sample dimensions for the different tests performed.

Sample size (*L* × *T* × *R*) (mm^3^)	Tests or analysisperformed	Number of samples per treatment
10 × 25 × 25	Physical properties, chemical characterisation, hygroscopic properties	10
50 × 50 × 25	Brinell hardness	15
180 × 10 × 10	Three points bending	30

### 2.2 Chemical characterization

#### 2.2.1 Fourier transform infrared spectroscopy

Fourier transform infrared (FTIR) spectroscopy was conducted on an Invenio S FTIR Spectrophotometer (Bruker Optik GmbH, Bremen, Germany) in the attenuated total reflection (ATR) mode to assess the chemical changes during the treatment. The measurements were performed at room temperature between wavenumbers of 4,000 and 400 cm^−1^ with a resolution of 4 cm^−1^. The samples were measured with 64 scans. Baseline corrections of the spectra were automatically performed by the baseline correction tool integrated in software. The peaks were normalized using the max-min normalization correction tool. Pieces of wood samples were cut using a clean blade and put on the diamond crystal for recording. For each kind of sample, two repetitions were recorded. Untreated, water-impregnated and cured at 160 °C, and 30 % CA-impregnated wood cured at different temperatures were measured.

#### 2.2.2 Solid-state ^13^C nuclear magnetic resonance spectroscopy

Solid-state ^13^C cross-polarization magic-angle spinning (CP/MAS) nuclear magnetic resonance (NMR) analysis was performed using a Bruker Avance III 400 spectrometer (Billerica, MA, USA) operated at 100 MHz. The MAS rate was set to 12 kHz, the sweep width to 29,761 Hz, the recycle delay to 2 s, and the CP contact to 2 ms. The chemical shifts were calibrated with the glycine carboxyl group at 176.03 ppm. All the spectra were normalized to the lignin band between 121 and 156 ppm for comparison. Untreated samples and 30 % CA impregnated samples dried before and after curing at different temperatures were measured and compared to complement FTIR results to better understand the chemical changes occurring during treatment.

### 2.3 Wood treatment and physical properties assessment

An aqueous solution of CA (30 % w/w) was prepared by dissolving 30 g of CA in 70 g of distilled water under stirring. The resulting solution density (*d*_sol_) was 1,130 ± 10 kg/m^3^. Wood samples were impregnated in an autoclave following these steps: a vacuum of 100 mbar was applied for 1 h, followed by a pressure of 11 bar for 2 h. Samples were taken out from the solution, and the excess solution was wiped off. They were weighed, and their dimensions were measured. The samples were dried at room temperature for seven days to avoid crack formation during curing. They were subjected to curing in a ventilated oven under atmospheric pressure at different temperatures: 100, 120, 140, and 160 °C for 24 h. The solution uptake (SU), weight percentage gain (WPG), cell wall bulking (CWB), and anti-swelling efficiency (ASE) before leaching were determined as described by Sèbe and Jéso (2000). The experimental SU of the 30 % (w/w) aqueous solution of CA was compared to the theoretical maximum uptake of the 30 % CA aqueous solution (SU_max_), the theoretical maximum water uptake (WU_max_), and the experimental uptake of water (WU) to assess the penetration of the solution inside the wood samples.

WU_max_ was calculated according to Equation (1) defined by Glass and Zelinka (2010).

(1) $${\text{WU}}_{\max}=\frac{{1.54-G}_{b}}{{1.54G}_{b}}\times 100$$

where 1.54 is the specific gravity of the wood cell wall, and *G*_b_ is the wood basic specific gravity:

$$G_{b}=\frac{m_{0}}{V_{1}}$$

The maximum possible solution uptake, SU_max_ was calculated according to Equation (2).

(2) $${\text{SU}}_{\max}=\left(\frac{V_{1}}{\;m_{0}\;}-\frac{1}{\;1.54}\right)\times d_{\text{sol}}\times 100$$

where *V*_1_ is the water-swollen wood volume, *m*_0_ is the oven-dry weight of unmodified wood, and *d*_sol_ is the density of the solution.

Here, it was assumed that the wood swelling by the solution is the same as by water, and the biomass occupies the portion corresponding to *m*_0_/1.54, which is not accessible to the solution volume. The remaining volume, *V*_1_ − (*m*_0_/1.54), is then assumed to be filled by the solution that keeps the bulk density *d*_sol_.

The water-leaching test followed European Committee for Standardization (CEN) (2020a), with the difference that the dry weight of the samples was determined after drying in an oven at 103 °C for at least 24 h, rather than in a climate chamber, as described in the standard. The WPG, CWB, and ASE were again determined and compared to their respective values before leaching. For all these determined properties, the mean and standard deviation were plotted.

### 2.4 Hygroscopic properties

To compare the effect of curing temperature on the hygroscopicity of wood treated with CA, the self-vapour absorption method was selected. Ten leached samples (10 × 25 × 25 mm^3^  *L* × *T* × *R*) of each treatment (untreated, water-treated (160 °C), and 30 % CA-treated at different temperatures) were placed in a desiccator above distilled water in a temperature-controlled room (21 °C ± 2). The samples were weighed over time. When the weight change was less than 1 % over 10 days, the moisture content at this time was used to calculate the moisture exclusion efficiency (MEE). The mean and corresponding standard deviation are presented in the results.

### 2.5 Mechanical properties

#### 2.5.1 Three-point bending test

The three-point bending tests and the Brinell hardness (BH) measurements were done using a table-top testing machine Z010 (ZwickRoell AG, Ulm, Germany). Three-point bending tests followed the European Committee for Standardization (CEN) (1978). Untreated, water-treated (160 °C), and 30 % CA impregnated and cured samples at different temperatures with dimensions of 180 × 10 × 10 mm^3^ (*L* × *T* × *R*) were analyzed. The samples were climatized at 20 °C and 65 % relative air humidity until constant weight. Prior to measurement, the precise thickness and width of the specimens were measured with a calliper (±0.01 mm). The load was applied in the tangential direction. The samples were placed on supports with a diameter of 15 mm, which were placed 150 mm apart (measured from the centre of the support). Force was applied with a stamp with diameter of 15 mm with a head speed of 2.5 mm/min. The modulus of elasticity was determined between an applied force of 50–150 N. The test was ended once a force reduction of 10 % occurred. The mean and standard deviation are presented in the results.

#### 2.5.2 Brinell hardness test

The BH test followed the European Committee for Standardization (CEN) (2020b). Fifteen samples per treatment were tested, and three points were measured on each sample. Untreated, water-treated (160 °C), and 30 % CA impregnated and cured at different temperatures with dimensions of 50 × 25 × 50 mm^3^ (*L* × *T* × *R*) were analyzed. The BH was measured on the tangential surface. The samples were climatized at 20 °C and 65 % relative air humidity until constant weight. Prior to measurement, the precise thickness of the specimens were measured with a calliper (±0.01 mm). A ball-shaped stamp with a diameter of 10 mm was used for the measurement. A preforce of 15 N was applied. For the test, the force was increased to 1 kN and held at this force for 25 s. The BH was calculated as described in the standard. The mean and standard deviation are presented in the results.

Equations used to determine the physical and mechanical properties are summarized in Supplementary Table S1.

### 2.6 Statistical analysis

All data were analyzed through R Studio (version 4.2.2 (2022), R Foundation for Statistical Computing, Vienna, Austria). A one-way analysis of variance (ANOVA) using the general linear model (GLM) procedure was conducted to assess the statistical significance of the differences. Before running the ANOVA test, the Shapiro-Wilk test was used to check if the data had a normal distribution. Rank transformation of the data was used when data did not present a normal distribution. The Kruskal-Wallis test was performed only when transformed data did not present a normal distribution. Differences between treatments were declared significant at *p* ≤ 0.05. A post-hoc comparison was done using the Tukey test to see which group pairs differed significantly.

## 3 Results and discussion

### 3.1 Chemical characterization

#### 3.1.1 Fourier transform infrared spectroscopy

**Figure 2:**
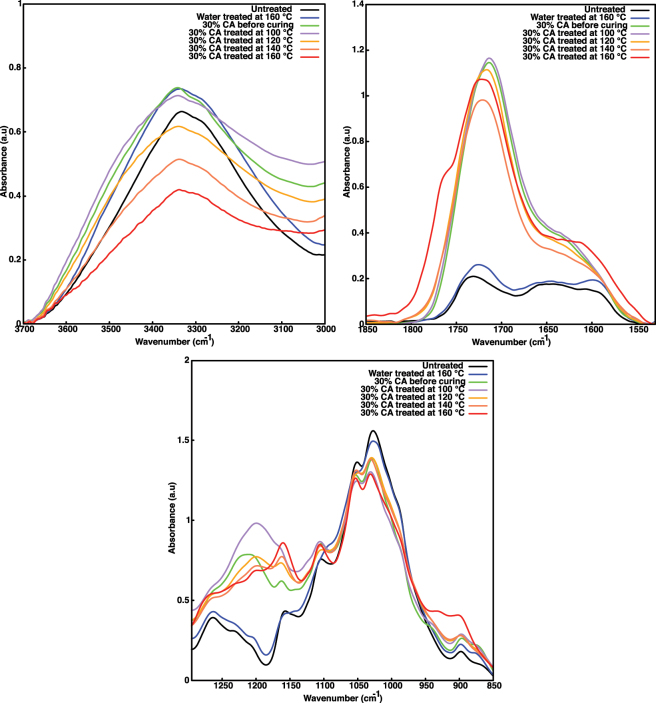
Comparison of FTIR spectra of untreated, water-modified (160 °C), and 30 % CA-modified at different temperatures. Zoom on three main regions where changes occur during modification.

FTIR comparison of untreated, water-treated (160 °C), CA impregnated and dried (uncured), and CA impregnated and cured at different temperatures wood is presented in Figure 2. Three main regions were chosen for the comparison. The hydroxy (OH) group region around 3,310 cm^−1^, as these groups react with CA (see Figure 1), the carbonyl group (C=O) region at 1,714 cm^−1^, and the carboxylate (C–O) region between 1,156 and 120 cm^−1^ were considered as representatives of the esterification reaction. The relative intensity of the OH group band decreased with an increase in temperature for 30 % CA-treated samples. The relative C=O intensity increased for CA-treated samples. At 160 °C, there was an appearance of a new band at 1,764 cm^−1^. For CA, this band is assigned to the anhydride form (Cai et al. 2019; Tsioptsias et al. 2024). The C–O carboxylate band of CA appeared at 1,200 cm^−1^ (Dong et al. 2012) after CA impregnation in wood. This band shifted to lower wavelengths when the temperature increased. These changes were not observed for the water-treated samples.

The decrease in the relative intensity of the OH group band suggests that increasing temperature enhances the reaction of CA with the OH groups in the wood cell wall matrix. The relative intensity of the C=O band increases during the treatment with CA, showing the occurrence of the esterification reaction within the wood (L’Hostis et al. 2018). Finally, the shift of the C–O carboxylate band to lower wavelengths suggests esterification between CA and the OH groups of the wood matrix (Kurkowiak et al. 2023). The melting point of CA is 156 °C, and the reaction of CA reaches its maximum around this temperature. Below 140 °C, the reaction between CA and OH groups of the wood matrix occurs to a low degree. The FTIR spectrum of the water-treated (160 °C) sample is similar to that of the untreated samples. Hence, the changes observed in 30 % CA-treated samples at 160 °C are mainly due to the CA reaction with the wood matrix.

#### 3.1.2 Solid-state ^13^C nuclear magnetic resonance spectroscopy

**Figure 3:**
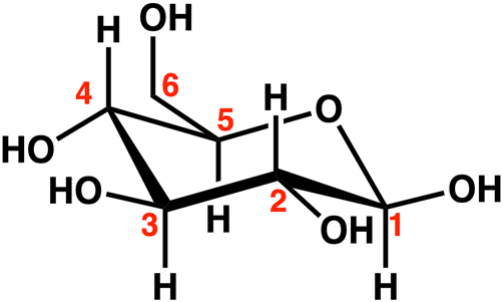
Carbon numbering in the molecular structure of glucose.

**Figure 4:**
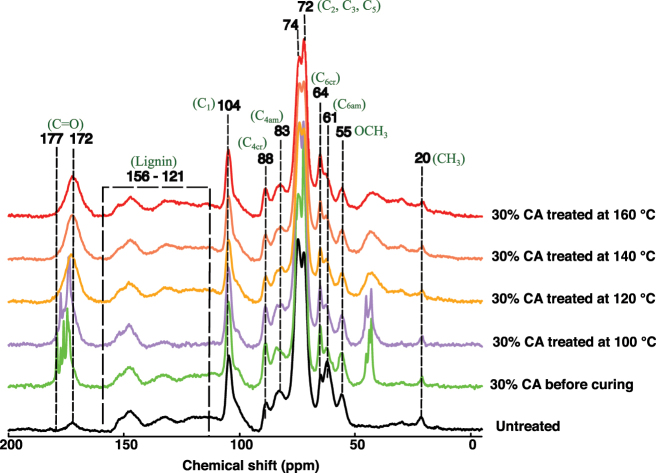
Comparison of ^13^C CP/MAS solid-state NMR spectra of wood samples treated with CA and different temperatures. C_1_–C_6_ correspond to cellulose and hemicellulose (see Figure 3); am, amorphous; cr, cristalline.

Untreated, 30 % CA impregnated and dry, and 30 % CA impregnated and cured at different temperatures wood samples were compared by solid-state ^13^C NMR analysis (Figure 4). All spectra were normalized to the wood lignin skeleton bands between 121 and 156 ppm (Santoni et al. 2015). Lignin was chosen as the reference for normalization due to its chemical stability compared to cellulose and hemicellulose during this treatment process. The spectrum of the wood sample impregnated with 30 % CA prior to curing (depicted in green) exhibits characteristic CA signals. The C=O bands of CA are observed at 174 and 177 ppm (Schantz et al. 2009). After post-curing at 100 °C of 30 % CA impregnated samples, no significant spectral changes are observed compared to the 30 % CA impregnated and dry without curing. In contrast, curing at temperatures ranging from 120 to 160 °C induces notable spectral alterations. Specifically, the C=O (acid) band shifts towards a lower chemical shift (∼170 ppm) with the increasing temperature. The intensity of the C_6_ of the crystalline cellulose in tg conformation (Figure 3), band at 64 ppm (carbon of primary alcohol) increased before curing when mixed with CA for these samples. Further changes are observed, including the reduction in intensity of the CH_3_ band of wood at 20 ppm when the temperature increased (Figure 4).

CA impregnation in wood without curing enhances cellulose crystallinity. This can be seen from the 61–64 ppm, and 83–88 ppm intensity ratios. However, the severity of the treatment leads to a decrease in the crystallinity, as evidenced by the intensity increase of the peak at 83 ppm. The peak at 61 ppm becomes lower. This peak corresponds to the surface of primary alcohol (C_6_) and might be an indication that surface hydroxy groups of cellulose are esterified to some extent, as seen in the FTIR analysis. Further, the shift of C=O band towards a lower chemical shift indicates the formation of the ester linkage and suggests that the formation of this linkage is heat-dependent. This shift aligns with an increase in the intensity of the C_6_ band, confirming a reaction between CA and the wood’s free OH groups within this temperature range (Noordover et al. 2007). The reduction in the intensity of the CH_3_ band of wood suggests the potential degradation of wood components at high temperature, as later observed by WPG.

### 3.2 Physical properties

#### 3.2.1 Solution uptake

**Figure 5:**
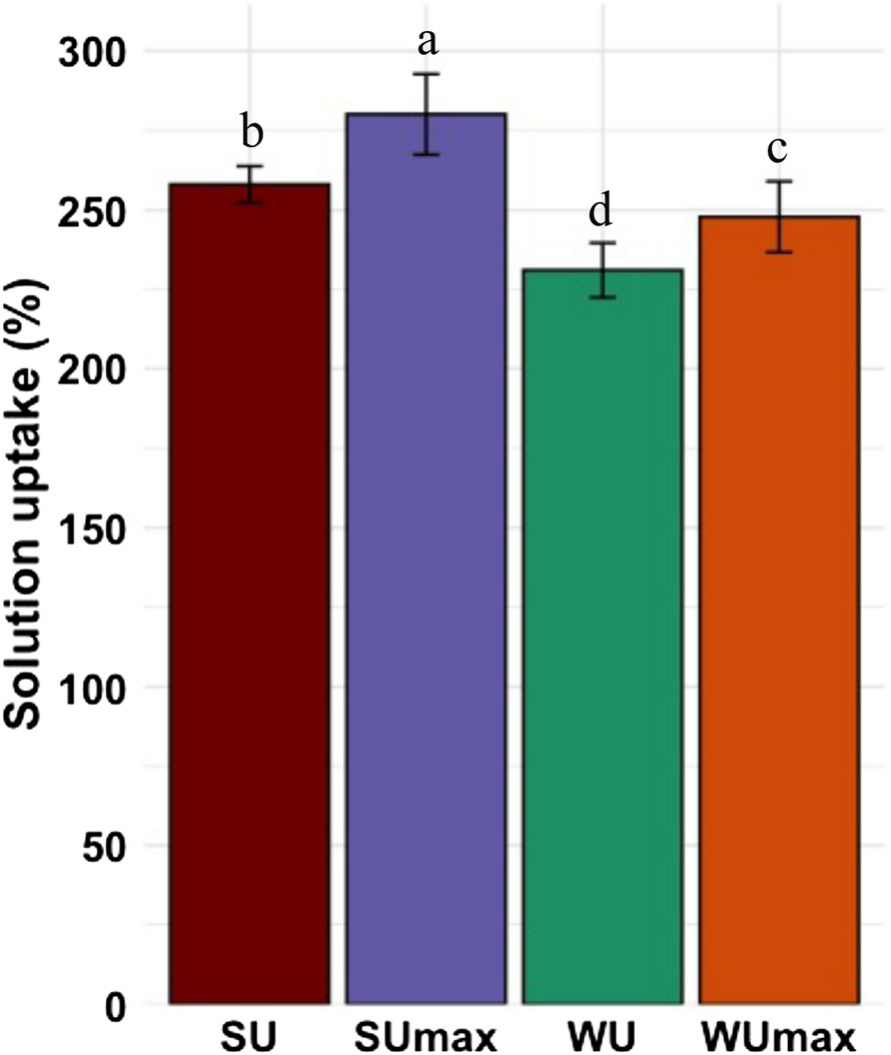
Comparison of the theoretical maximum water uptake (WU_max_), theoretical maximum 30 % CA solution uptake (SU_max_), experimental 30 % CA solution uptake (SU), and experimental water uptake (WU) by Scots pine sapwood. Different superscript letters indicate a significant difference between solutions at *p* ≤ 0.05 level.

WU_max_ and SU_max_ are the ideal situations in which all the void volume in wood is filled with water and 30 % CA solution, respectively. WU and SU were the measured water uptake and 30 % CA solution uptake by wood samples, respectively. The SU of Scots pine sapwood samples impregnated with 30 % CA solution was compared to SU_max_ (Figure 5). The SU_max_ was higher than SU, which in turn was higher than WU_max_, which was higher than WU. They were all above 250 % except WU.

The highest uptake was observed for SU_max_, as expected, since it accounts for complete saturation under ideal conditions, whereas SU would leave some void volume in wood inaccessible to the CA solution during the impregnation process. However, the experimental uptake (SU) remained high, approximately 90 % of SU_max_, suggesting good permeability and efficient penetration of the CA solution into the wood matrix. The reduced WU_max_ compared to SU_max_ can be attributed mainly to the higher density of the CA solution compared to water. Additional factors leading to the differences in the solution uptakes could be the viscosity and surface tension. Moreover, the difference between SU and SU_max_ suggests that under experimental conditions, full saturation may not be achieved due to localized variations in permeability, anatomical heterogeneity, or solution viscosity effects. The SU obtained herein is slightly higher than the one obtained by Hötte and Militz (2024) during the impregnation of the same sample with 30 % CA. The discrepancy comes from the fact that the authors calculated SU from the climatized weight, and in this study, it was calculated from the dry weight.

#### 3.2.2 Comparison of the weight percentage gain before and after the leaching test (European Committee for Standardization (CEN) 2020a)

**Figure 6:**
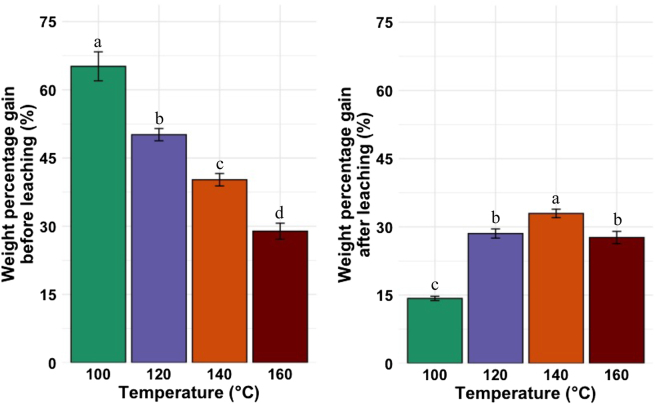
Comparison of weight percentage gain before and after the leaching test on wood samples treated with a 30 % (w/w) aqueous solution of CA at different temperatures. Different superscript letters indicate a significant difference between the curing temperatures at *p* ≤ 0.05 level.

The WPG of all treated wood before and after the leaching test (European Committee for Standardization (CEN) 2020a) were compared (Figure 6, left and right, respectively). Before the leaching test, the WPG significantly decreased (*p* ≤ 0.05) with an increase in the curing temperature. However, after the leaching test, the WPG increased with increasing curing temperature up to 140 °C, then decreased significantly above 140 °C.

**Figure 7:**
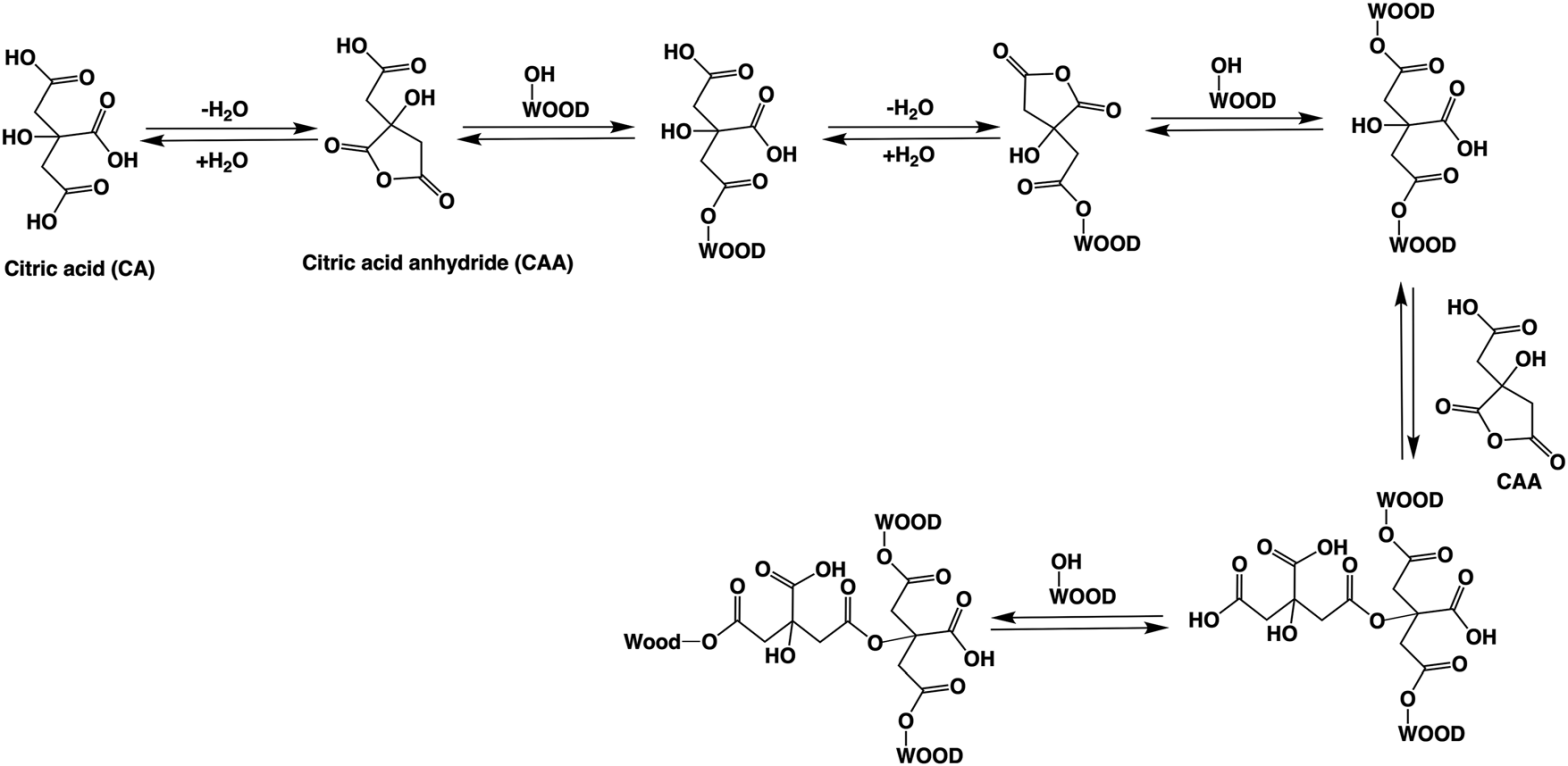
Potential scenario that could happen during wood modification with CA.

A SU of 250 % of 30 % of CA should result in 75 % of WPG after water evaporation, but the 100 °C WPG is a bit lower (65 %). One possible reason is the slightly selective uptake of water from the CA solution due to its higher mobility. The low temperature would not allow for a complete conversion of CA to CA anhydride (CAA), which could subsequently react with wood hydroxy groups (wood esterification). Hence, these results suggest that below 140 °C, the energy is insufficient for complete CA conversion to CAA, which can then further react with the OH groups of the wood cell wall matrix. This is why, after the leaching test, the WPG is significantly decreased due to unreacted CA molecules that are easily leachable by water. At 140 °C, there is also a decrease in WPG after the leaching test, but the WPG at 140 °C remains the highest compared to other temperatures. At 160 °C, the WPG values before and after the leaching of the leaching test are similar; they are lower compared to those at 140 °C, both before and after the leaching test. This suggests that at higher temperatures (160 °C), there is an acidic thermal degradation of the wood cell wall components. The results of water treatment and water + HCl support this hypothesis (Supplementary Table S2). The samples cured at 160 °C after impregnation with water + HCl exhibited a weight loss higher than 4.5 %, while those impregnated with water presented a weight loss inferior to 2.5 %. Another hypothesis that could explain this is the thermal degradation of CA into volatile compounds at higher temperatures (Supplementary Table S3). However, higher temperature promotes a higher degree of CA condensation on wood cell wall components, as observed by the WPG after the leaching test (Figure 6, right). L’Hostis et al. (2018) observed a better fixation of CA to wood when the temperature increased. A probable auto-reaction of CA could also happen, as presented in Figure 7, but with these results, it is difficult to prove.

#### 3.2.3 Cell wall bulking before and after the leaching test (European Committee for Standardization (CEN) 2020a)

**Figure 8:**
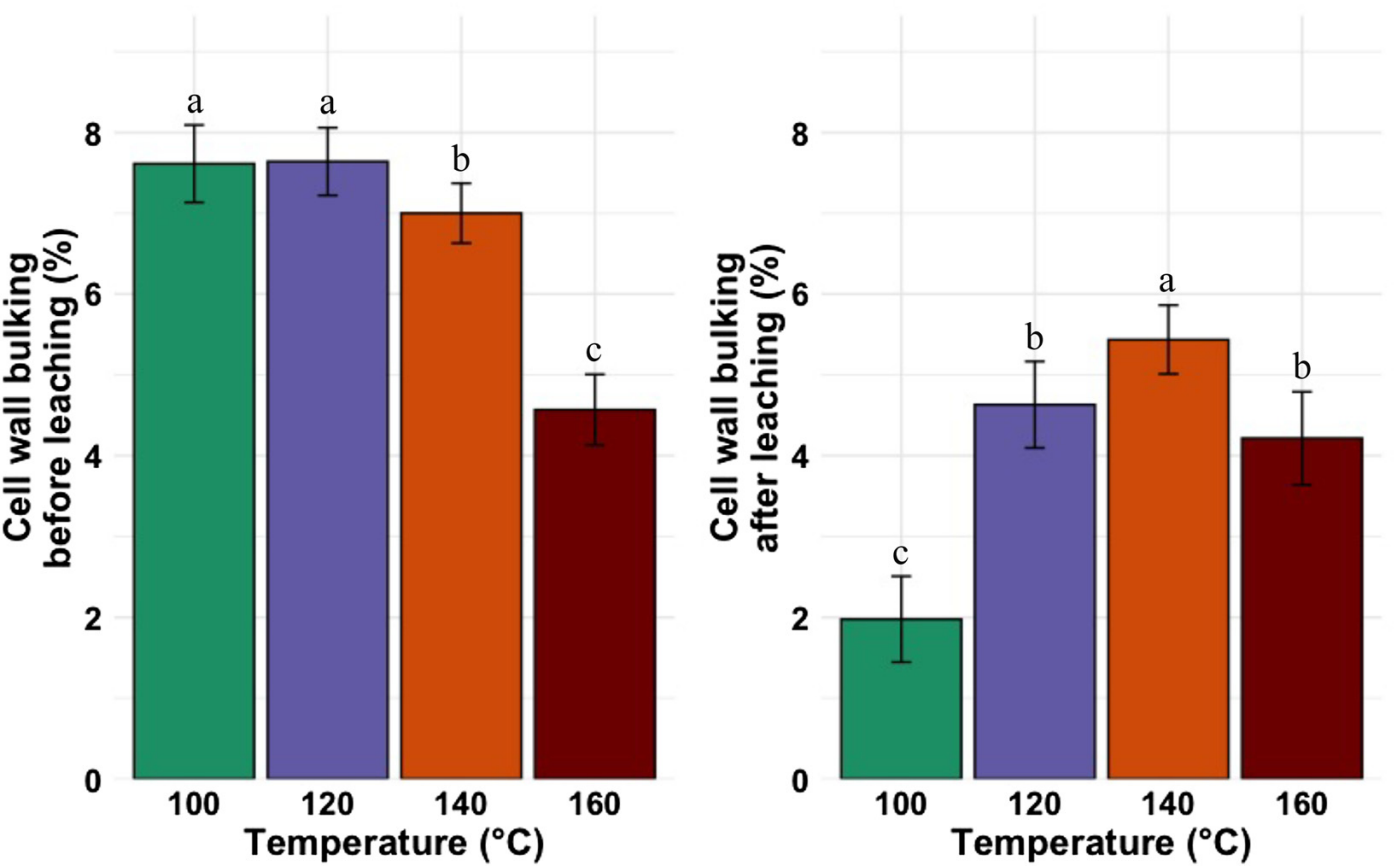
Comparison of cell wall bulking before and after the leaching test of wood samples treated with 30 % (w/w) aqueous solution of CA at different temperatures. Different superscript letters indicate a significant difference between the curing temperatures at *p* ≤ 0.05 level.

The CWB before the leaching test shows a descending trend with the curing temperature, especially decreasing significantly (*p* ≤ 0.05) from 140 °C (Figure 8, left). After the leaching test, the CWB (Figure 8, right) followed the WPG trend after the leaching test; the CWB significantly (*p* ≤ 0.05) increased with temperature increasing up to 140 °C, then decreased.

Before the leaching test, the higher values of CWB observed at temperatures below 140 °C are explained by the unreacted CA present in the cell wall. The lower values of CWB at temperatures above 140 °C can be explained by the hydrolysis of cell wall components. After the leaching, the decrease in the CWB values at temperatures below 140 °C is explained by the fact that CA reacts with the OH groups of the wood cell wall matrix, but to a lower degree, and part of CA remains unreacted and leaches out. Above 140 °C, the temperature and the acid medium lead to the hydrolysis of the wood components. The low loss in CWB at 160 °C suggests that the CA fraction is completely condensed on wood cell wall components.

#### 3.2.4 Anti-swelling efficiency before and after the leaching test (European Committee for Standardization (CEN) 2020a)

**Figure 9:**
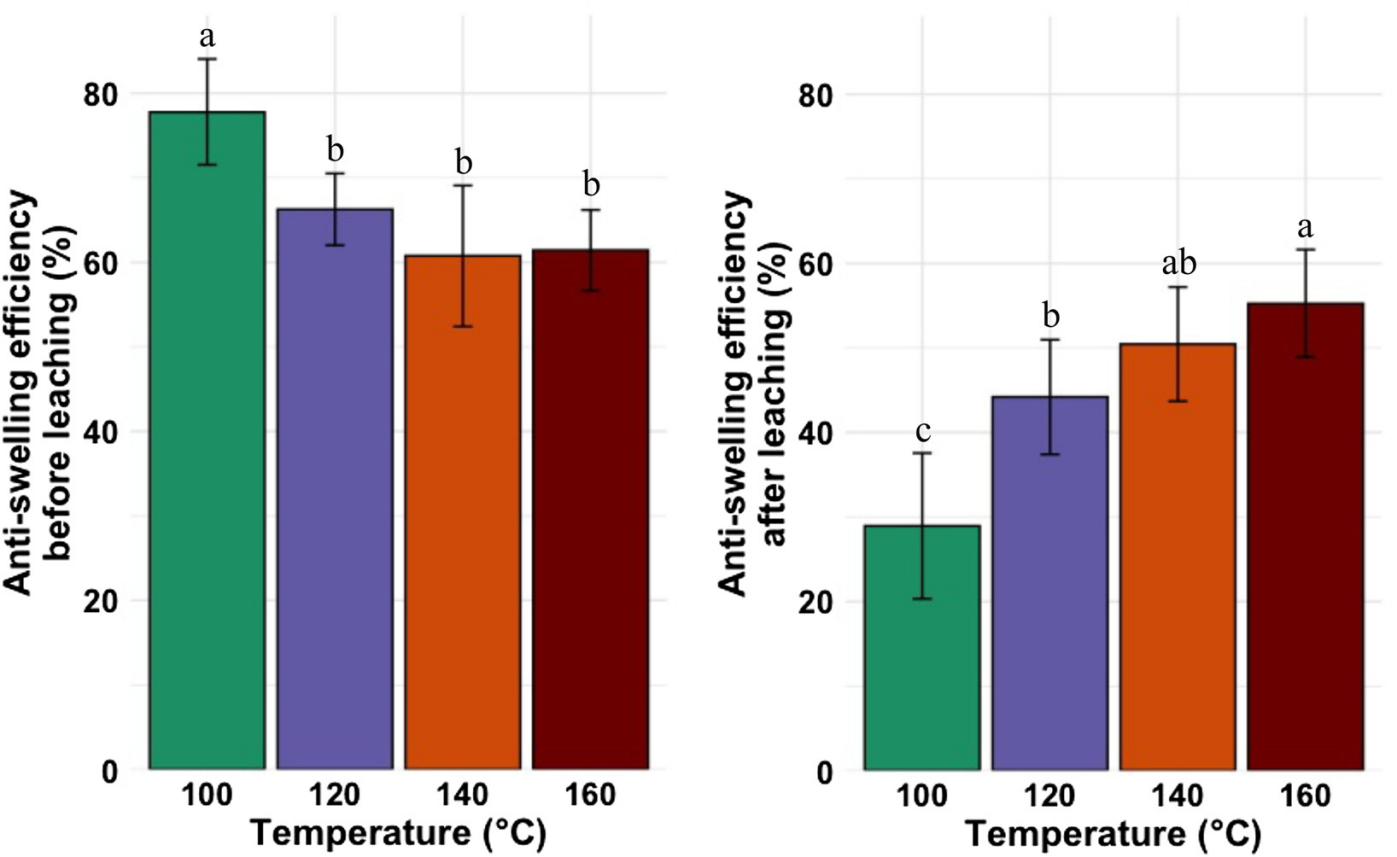
Comparison of anti-swelling efficiency before and after the leaching test of wood samples treated with 30 % (w/w) aqueous solution of CA at different temperatures. Different superscript letters indicate a significant difference between the curing temperatures at *p* ≤ 0.05 level.

Before the leaching test, all samples exhibited an ASE over 55 %. The ASE values for samples treated at 120, 140, and 160 °C were similar (*p* ≥ 0.05), and they were higher (*p* ≤ 0.05) than those of samples cured at 100 °C (Figure 9, right). After the leaching test, ASE values increased with increasing temperature (Figure 9, left). The highest value was measured for samples cured at 160 °C, and the lowest value for the samples cured at 100 °C.

Several theories explained wood dimensional stability by means of wood modification. First, the wood cell wall expansion to its maximum by the impregnated agents (Hill 2006), second, the reduction of wood moisture uptake sites by the reaction with impregnated agents (Rowell 2014), and finally, the lignin modification (crosslinking), which prevents moisture absorption (Esteves and Pereira 2009). CA acts as a binding agent and is well known for increasing wood dimensional stability. In this study, the loss in WPG and CWB after the leaching test was explained by the low degree of CA reaction with the OH group of the wood cell wall matrix (reduction of moisture uptake sites) below 140 °C. L’Hostis et al. (2018) observed an increase in ASE with an increase in temperature when modifying beech wood with CA. The highest ASE values observed at 160 °C confirm the high degree of CA reaction with OH groups of the wood matrix. However, based on those results alone, it is difficult to identify the cause of this effect among the three possible scenarios mentioned above. It could be a combination of the three effects.

#### 3.2.5 Moisture exclusion efficiency

The MEE of wood samples subjected to different treatments is presented in Table 2. MEE represents the water repellence capacity of treated samples compared to untreated samples. The results indicate a clear influence of CA modification and treatment temperature on the wood’s hygroscopic behavior. The water-treated (160 °C) exhibited the lowest MEE (13 ± 2 %). Wood samples treated with 30 % CA showed a progressive increase in MEE as the treatment temperature increased from 100 to 160 °C. The MEE rose from 16 ± 1 % at 100 °C to a maximum of 41 ± 4 % at 160 °C.

**Table 2:** Comparison of the moisture exclusion efficiency of water-treated (160 °C) and 30 % CA cured wood samples at different temperatures.

Treatment	Moisture exclusion efficiency (%)
Water-treated (160 °C)	13 ± 2^e^
30 % CA 100 °C	16 ± 1^d^
30 % CA 120 °C	19 ± 2^c^
30 % CA 140 °C	30 ± 4^b^
30 % CA 160 °C	41 ± 4^a^

Different superscript letters indicate a significant difference between the curing temperatures at *p* ≤ 0.05 level.

The lowest value of MEE for water-treated (160 °C) samples confirms that heat (160 °C) has a minor impact on reducing moisture uptake during this process. The results with CA treatment suggest that elevated temperatures enhance esterification between CA and wood cell wall components, resulting in a greater reduction in hygroscopicity. The main mechanism behind the MEE decrease is the reaction of CA with free OH groups within the wood matrix. This phenomenon is well documented in the literature, where ester bond formation decreases accessible hydroxy groups for water sorption (Awais et al. 2022; Popescu et al. 2014; Xie et al. 2010). It is also noteworthy that 30 % CA-treated samples cured at a higher temperature (160 °C) benefited from the additional effect of heat, as their MEE was the highest despite showing similar WCB to samples cured at 120 °C. This mechanism is supported by the concurrent increase in WPG observed for similar treatment conditions (refer to Figure 2). The MEE increase observed between 140 and 160 °C suggests that reaction completeness and crosslink density may continue to develop at higher temperatures. Overall, these findings demonstrate that CA treatment is a promising approach for improving the dimensional stability of wood by reducing its hygroscopicity. These findings align with previous studies demonstrating the role of esterification in improving dimensional stability (Anwar Uyup et al. 2021; Donath et al. 2004; Rowell 2014).

### 3.3 Mechanical properties

#### 3.3.1 Three points bending test

**Figure 10:**
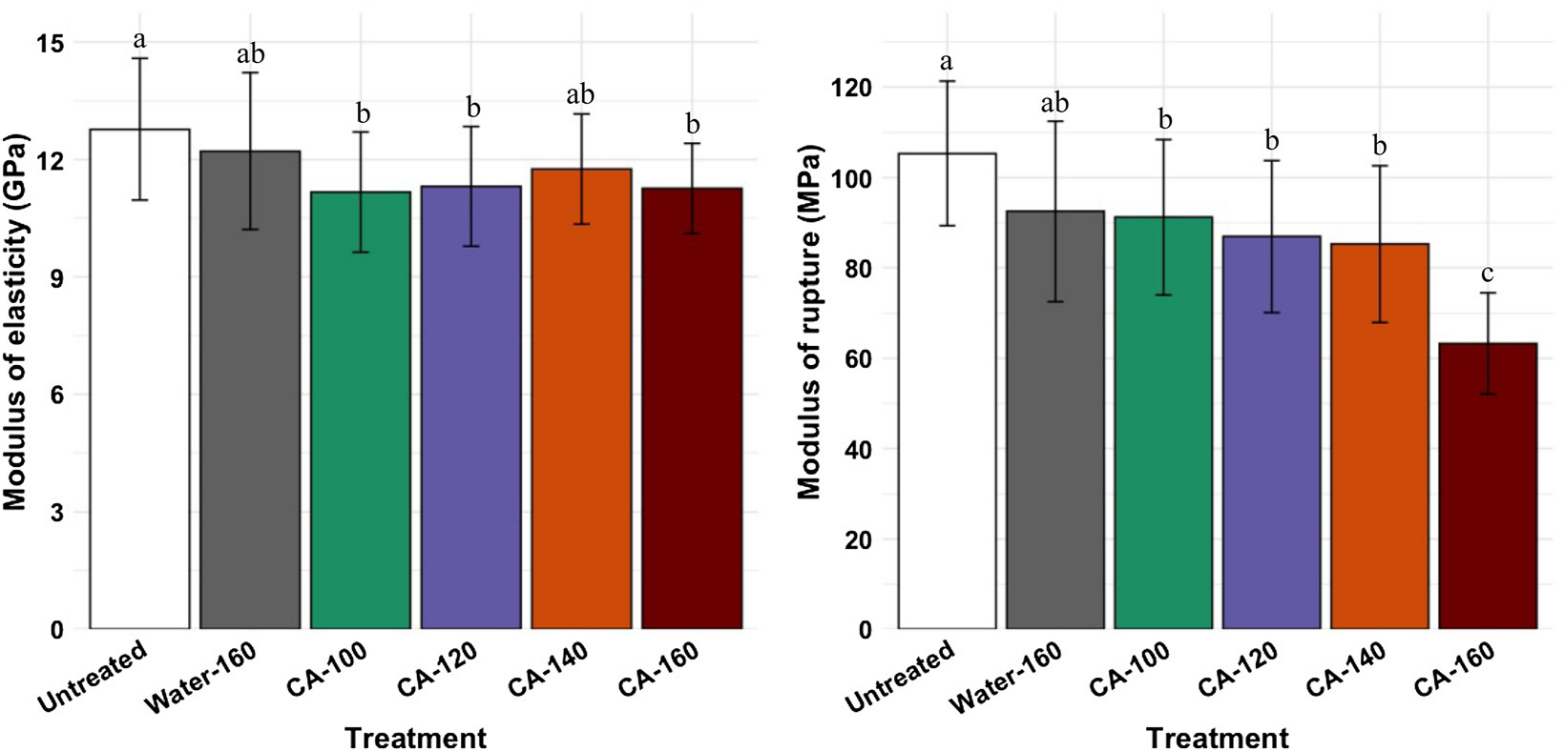
Comparison of modulus of elasticity and modulus of rupture of untreated, water-treated (160 °C) and wood samples treated with 30 % (w/w) aqueous solution CA at different temperatures. Different superscript letters indicate a significant difference between treatments at *p* ≤ 0.05 level.

The wood treatment with CA decreased its bending strength (Figure 10). The modulus of elasticity (MOE) of untreated wood samples was not significantly different from that of the samples impregnated with water and cured at 160 °C. However, it was higher than that of samples impregnated with 30 % CA and cured from 100 to 160 °C (Figure 10, left). The modulus of rupture (MOR) showed a substantial decrease between untreated and treated samples (Figure 10, right). The water-treatment (160 °C) resulted in a 10–20 % decrease in MOR. Samples impregnated with 30 % CA and cured at 100, 120, and 140 °C presented similar MOR. Samples impregnated with 30 % CA and cured at 160 °C lost 42 % of their MOR compared to that of untreated samples.

The wood modification with CA is well-known to decrease its bending strength (Augustina et al. 2023; Dong et al. 2023; Feng et al. 2014; L’Hostis et al. 2018). The decrease in MOR is explained by the reaction of CA with the adjacent cellulose fibers, which restricts the cell wall movement and makes wood brittle. However, the difference between samples cured at different temperatures is explained by the degree of esterification reaction and the hydrolysis catalyzed by the acidic conditions. Curing CA-impregnated wood at 160 °C allows a high degree of reaction between CA and free OH groups of the wood matrix.

#### 3.3.2 Brinell hardness

**Figure 11:**
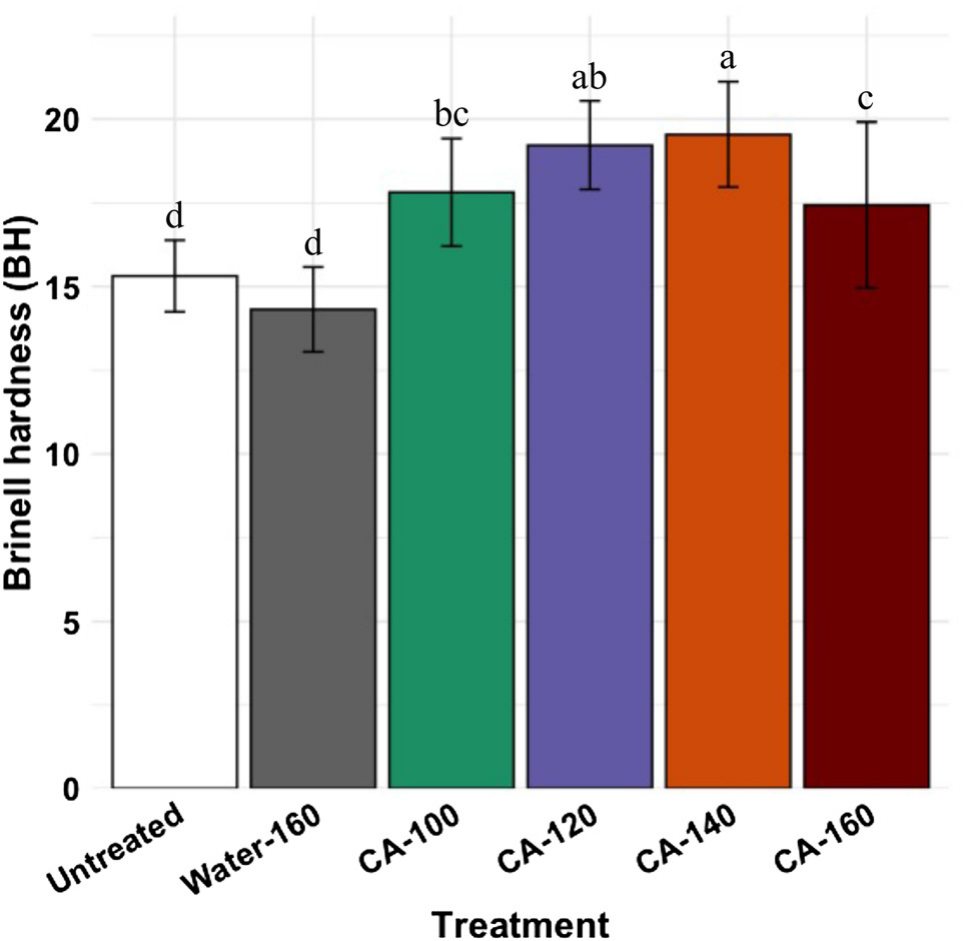
Comparison of Brinell hardness of untreated, water-treated (160 °C), and wood samples treated with 30 % (w/w) aqueous solution CA at different temperatures. Different superscript letters indicate a significant difference between treatments at *p* ≤ 0.05 level.

The BH of water-treated samples was not significantly different than that of untreated samples. However, there was an increase in BH with an increase in temperature for samples impregnated with 30 % CA and cured at different temperatures. The BH increased until 140 °C, then decreased (Figure 11). Under these conditions (140 °C, 24 h), the BH was increased by 33 % compared to that of untreated samples.

The water treatment (160 °C) slightly decreases the hardness compared to other treated wood specimens, as this treatment removes part of the cell wall components. The BH increases for 30 % CA treated samples as they gain density after treatment (Supplementary Figure S1). The hardness of samples cured at 160 °C is lower than that of samples cured at 140 °C. This is explained by the degradation of wood components under these conditions (160 °C + acid medium).

## 4 Conclusions

This study demonstrates that temperature plays a decisive role in determining the balance between improved dimensional stability and mechanical strength in CA-modified wood. While treatments above 140 °C promote stronger esterification between CA and the wood matrix, as confirmed by FTIR and NMR analyses, as well as the low leaching rate after the EN 84 test, they also accelerate wood degradation and reduce bending strength. Conversely, treatments at lower temperatures preserved mechanical integrity but yielded less improvement in dimensional stability as the degree of CA reaction with wood is lower. The best compromise in this study in terms of dimensional stability and mechanical properties of the wood is a curing at 140 °C for 24 h. These results highlight the necessity of carefully optimizing processing conditions to achieve durable yet mechanically reliable CA-treated wood. From an application standpoint, this work provides insight into developing industrially viable CA-based modification processes that minimize brittleness while leveraging CA’s environmental advantages. Future research could focus on determining the optimal kinetics of the CA reaction within wood, as well as its activation energy, to improve wood’s properties.

## Supplementary Material

Supplementary Material

Supplementary Material

Supplementary Material

Supplementary Material

## References

[j_hf-2025-0127_ref_001] Anwar Uyup M.K., Sahari S.H., Jalaludin Z., Husain H., Lee S.H., Yusoh A.S. (2021). Water vapour sorption behaviour and physico-mechanical properties of methyl methacrylate (MMA)- and MMA–styrene-modified batai (*Paraserianthes falcataria*) wood. *Holzforschung*.

[j_hf-2025-0127_ref_002] Apelblat A. (2014). *Citric acid*.

[j_hf-2025-0127_ref_003] Augustina S., Marbun S.D., Sudarmanto N., Trisatya D.R., Santoso E.B., Pramadani D., Afni N.N., Farih T.A., Tobing G.W.L., Syafi’i W. (2023). Dimensional stability and mechanical properties of citric acid impregnated samama wood (*Anthocephalus macrophyllus* (Roxb) Havil) at high curing temperatures. *J. Korean Wood Sci. Technol.*.

[j_hf-2025-0127_ref_004] Awais M., Altgen M., Belt T., Teräväinen V., Mäkelä M., Altgen D., Nopens M., Rautkari L. (2022). Wood–water relations affected by anhydride and formaldehyde modification of wood. *ACS Omega*.

[j_hf-2025-0127_ref_005] Bischof Vukusic S., Katovic D., Schramm C., Trajkovic J., Sefc B. (2006). Polycarboxylic acids as non-formaldehyde anti-swelling agents for wood. *Holzforschung*.

[j_hf-2025-0127_ref_006] Cahyono T.D., Syahidah (2019). Citric acid, an environmentally friendly adhesive and wood impregnation material-review of research. *IOP Conf. Ser.: Mater. Sci. Eng.*.

[j_hf-2025-0127_ref_007] Cai Z., Ji B., Yan K., Zhu Q. (2019). Investigation on reaction sequence and group site of citric acid with cellulose characterized by FTIR in combination with two-dimensional correlation spectroscopy. *Polymers*.

[j_hf-2025-0127_ref_008] Ciriminna R., Meneguzzo F., Delisi R., Pagliaro M. (2017). Citric acid: emerging applications of key biotechnology industrial product. *Chem. Cent. J.*.

[j_hf-2025-0127_ref_009] Cruz-Lopes L., Sell M., Lopes R., Esteves B. (2025). Enhancing Pinus pinaster wood durability through citric acid impregnation. *Sustainability*.

[j_hf-2025-0127_ref_012] Donath S., Militz H., Mai C. (2004). Wood modification with alkoxysilanes. *Wood Sci. Technol.*.

[j_hf-2025-0127_ref_013] Dong Y., Shao J., Chen C., Li H., Wang R., Chi Y., Lin X., Chen G. (2012). Blue luminescent graphene quantum dots and graphene oxide prepared by tuning the carbonization degree of citric acid. *Carbon*.

[j_hf-2025-0127_ref_014] Dong Y., Xue Q., Fu Z., Yan Y., Lu Y., Liu Y., Li J. (2023). Enhancing wood stability and fire retardancy through citric acid and phosphorylated sucrose stearate cross-linking modification. *Constr. Build. Mater.*.

[j_hf-2025-0127_ref_016] Esteves B., Pereira H. (2009). Wood modification by heat treatment: a review. *Bioresources*.

[j_hf-2025-0127_ref_010] European Committee for Standardization (CEN) (1978). ..

[j_hf-2025-0127_ref_015] European Committee for Standardization (CEN) (2020a). *Durability of wood and wood-based products – Accelerated ageing of treated wood prior to biological testing – Leaching procedure* (DIN EN 84:2020-10). ..

[j_hf-2025-0127_ref_011] European Committee for Standardization (CEN) (2020b). *Wood flooring and parquet - Determination of resistance to indentation -*.

[j_hf-2025-0127_ref_017] Feng X., Xiao Z., Sui S., Wang Q., Xie Y. (2014). Esterification of wood with citric acid: the catalytic effects of sodium hypophosphite (SHP). *Holzforschung*.

[j_hf-2025-0127_ref_018] Glass S., Zelinka S. (2010). Moisture relations and physical properties of wood. *Wood handbook: wood as an engineering material*.

[j_hf-2025-0127_ref_019] Hill C.A.S. (2006). Wood modification: chemical, thermal and other processes.

[j_hf-2025-0127_ref_020] Hötte C., Militz H. (2024). Esterification of wood with citric acid and sorbitol: effect of the copolymer on the properties of the modified wood. Part 1: macroscopic changes, fixation of chemicals and impact bending properties. *Holzforschung*.

[j_hf-2025-0127_ref_021] Kurkowiak K., Hentges D., Dumarçay S., Gérardin P., Militz H. (2023). Understanding the mode of action of sorbitol and citric acid (SorCA) in wood. *Wood Mater. Sci. Eng.*.

[j_hf-2025-0127_ref_022] Lee S.H., Tahir P.M., Lum W.C., Tan L.P., Bawon P., Park B.-D., Edrus S.S.O.A., Abdullah U.H. (2020). A review on citric acid as green modifying agent and binder for wood. *Polymers*.

[j_hf-2025-0127_ref_023] L’Hostis C., Thévenon M.-F., Fredon E., Gérardin P. (2018). Improvement of beech wood properties by *in situ* formation of polyesters of citric and tartaric acid in combination with glycerol. *Holzforschung*.

[j_hf-2025-0127_ref_024] Noordover B.A.J., Duchateau R., Van Benthem R.A.T.M., Ming W., Koning C.E. (2007). Enhancing the functionality of biobased polyester coating resins through modification with citric acid. *Biomacromolecules*.

[j_hf-2025-0127_ref_025] Popescu C.-M., Hill C.A.S., Curling S., Ormondroyd G., Xie Y. (2014). The water vapour sorption behaviour of acetylated birch wood: how acetylation affects the sorption isotherm and accessible hydroxyl content. *J. Mater. Sci.*.

[j_hf-2025-0127_ref_026] Rowell R.M. (2014). Acetylation of wood – a review. *Int. J. Lignocell. Prod.*.

[j_hf-2025-0127_ref_027] Santoni I., Callone E., Sandak A., Sandak J., Dirè S. (2015). Solid state NMR and IR characterization of wood polymer structure in relation to tree provenance. *Carbohydr. Polym.*.

[j_hf-2025-0127_ref_028] Schantz S., Hoppu P., Juppo A.M. (2009). A solid-state NMR study of phase structure, molecular interactions, and mobility in blends of citric acid and Paracetamol. *J. Pharm. Sci.*.

[j_hf-2025-0127_ref_029] Schramm C., Rinderer B. (1999). Influence of additives on the formation of unsaturated PCAs produced during durable‐press curing with citric acid. *Color. Technol.*.

[j_hf-2025-0127_ref_030] Sèbe G., Jéso B.D. (2000). The dimensional stabilisation of maritime pine sapwood (*Pinus pinaster*) by chemical reaction with organosilicon compounds. *Holzforschung*.

[j_hf-2025-0127_ref_031] Tsioptsias C., Panagiotou A., Mitlianga P. (2024). Thermal behavior and infrared absorbance bands of citric acid. *Appl. Sci.*.

[j_hf-2025-0127_ref_032] Xie Y., Hill C.A.S., Xiao Z., Jalaludin Z., Militz H., Mai C. (2010). Water vapor sorption kinetics of wood modified with glutaraldehyde. *J. Appl. Polym. Sci.*.

