# Supplementary material for: Effect of temperature on wood modification with citric acid
Source: Holzforschung. 2026 Jan 19;80(3):231–42. doi: 10.1515/hf-2025-0127 (PMC12958830; doi:10.1515/hf-2025-0127)
Supplement: Supplementary file 4 — Supplementary Material [file j_hf-2025-0127_suppl_004.docx]

**Supplementary Material**

**Effect of temperature on wood modification with citric acid**

Assira Keralta et al.

DOI 10.1515/hf-2025-0127


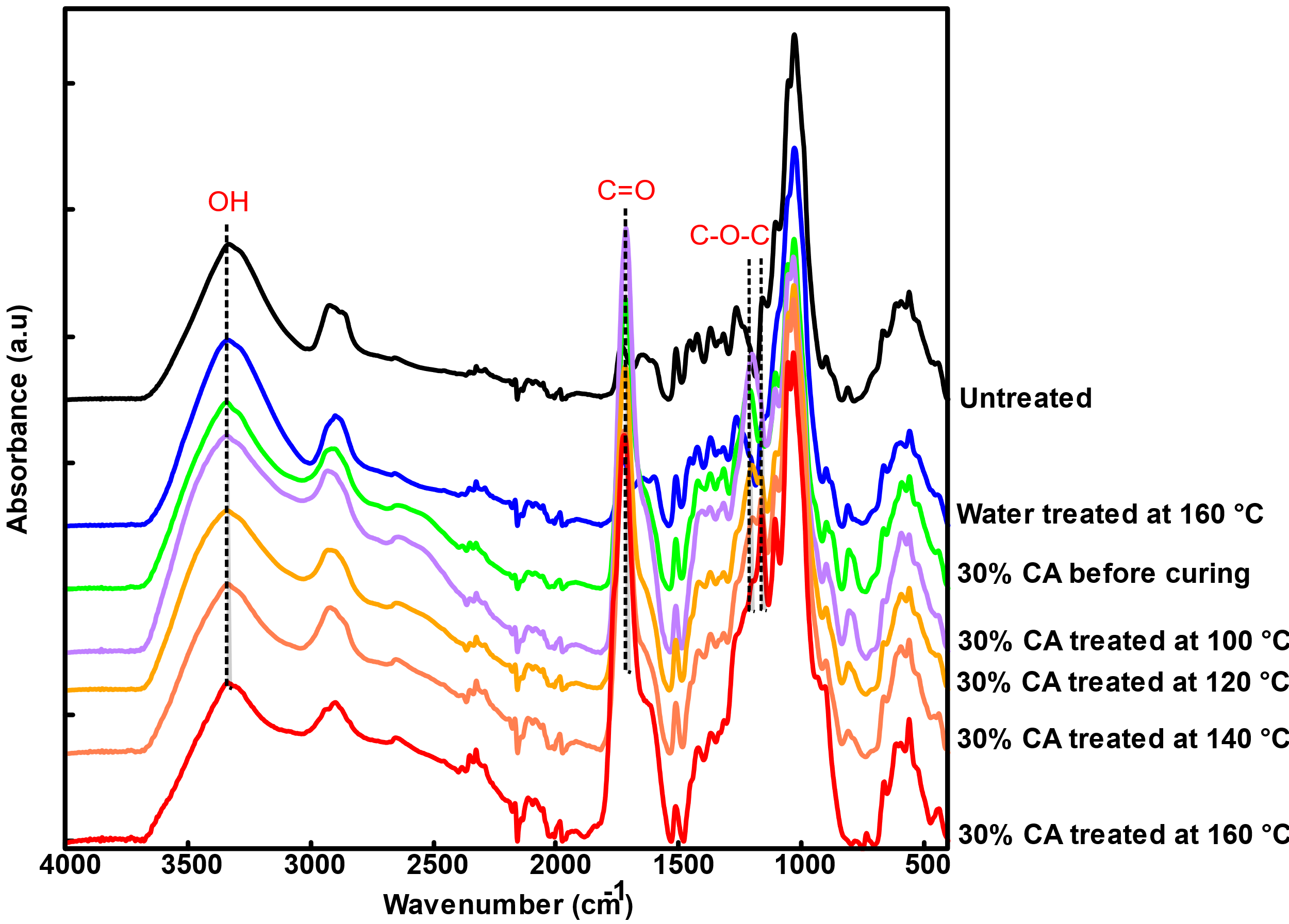


**Supplementary Figure S1:** Entire FTIR spectra comparison of untreated, water-treated (160 °C), and 30% CA‑treated wood at different temperatures.

**Supplementary Table S1:** The equations used to determine physical and mechanical properties.

| **Measured properties** | **Equation used for the calculation** | **Assignments of parameters** |
| --- | --- | --- |
| Solution uptake (*SU*) | $SU=\frac{m_{1}-m_{0}}{m_{0}}\text{×}\text{ }100$ | *m_0_*: oven-dry weight before treatment  *m_1_*: weight after impregnation |
| Maximum solution uptake (*SU_max_*) | ${SU}_{max}=(\frac{V_{1}}{m_{0}}-\frac{1}{1.54}\text{) ×} d_{sol}\text{ }\text{×} 100$ | *d_sol_*: density of the solution |
| Maximum water uptake (*WU_max_*) | $WUmax =\frac{{1.54-G}_{b}}{1.54 G_{b}} \text{×} 100$ | *G_b_*: wood specific density |
| Weight percentage gain before leaching (*WPG)* | $WPG=\frac{m_{2}-m_{0}}{m_{0}}\text{ }\text{×}100$ | *m_2_*: oven-dry weight after curing at 160 °C |
| Weight percentage gain after leaching (*WPG'*) | $WPG'=\frac{m_{3}-m_{0}}{m_{0}}\text{×}\text{ }100$ | *m_3_*: oven-dry weight after leaching |
| Cell wall bulking before leaching (*CWB*) | $CWB=\frac{A_{1}-A_{0}}{A_{0}}\text{×}\text{ }100$ | *A_0_*: oven-dry cross-sectional area before treatment  *A_1_*: oven-dry cross-sectional area after curing at 160 °C. |
| Cell wall bulking after leaching (*CWB'*) | $CWB'=\frac{A_{2}-A_{0}}{A_{0}}\text{×}\text{ }100$ | *A_2_*: oven-dry cross-sectional area after leaching |
| Volumetric swelling (*S*) | $S=\frac{V_{1}-V_{0}}{V_{0}}\text{×}\text{ }100$ | *V_0_*: oven-dry volume *V_1_*: volume after soaking |
| Anti-swelling efficiency before leaching (*ASE*) | $ASE=\frac{S_{0}-S_{1}}{S_{0}}\text{×}\text{ }100$ | *S_0_*: volumetric swelling before treatment *S_1_*: volumetric swelling after curing at 160 °C |
| Anti-swelling efficiency after leaching (*ASE'*) | $ASE'=\frac{S_{0}-S_{2}}{S_{0}}\text{×}\text{ }100$ | *S_2_*: volumetric swelling after leaching |
| Moisture content (*MC*) | $MC=\frac{m_{t}-m_{0}}{m_{0}}\text{×}\text{ }100$ | *m_t_*: weight during conditioning in desiccator at time t |
| Moisture exclusion efficiency (*MEE*) | $MEE=\frac{{EMC}_{0}-{EMC}_{t}}{{EMC}_{0}}\text{×}\text{ }100$ | *EMC_0_*: equilibrium moisture content before treatment  *EMC_t_*: equilibrium moisture content after treatment |
| Modulus of elasticity (*MOE*) | $MOE=\frac{L^{3}}{4 \text{×}b \text{×}h^{3}} \text{×}\text{ }\frac{DF}{Df}$ | *F*: force  f*:* strain  *L*: support span  *b*: width  *h*: thickness |
| Modulus of rupture (*MOR*) | $MOR=\frac{3 \text{×}F \text{×}L}{2 \text{×}b \text{×}h^{2}}$ |  |
| Brinell hardness (*BH*) | $BH=\frac{2F}{\left( \pi\cdot D\cdot\left( D-\sqrt{D^{2}-d^{2}} \right) \right)}$ | *F*: Force  *D*: diameter of the ball  *d*: diameter of the residual impression |
|  | $d=2\sqrt{h(D-h)}$ | *h*: depth |

**Supplementary Table S2:** Comparison of the weight loss percentage of wood samples cured at 160 °C after impregnation with water and water + HCl (160 °C).

| **Treatment** | **Weight loss percentage (%)** | |
| --- | --- | --- |
|  | **Mean** | **Standard deviation** |
| Water | 2.25 | 0.38 |
| *Water + HCl (160 °C) | 4.64 | 0.40 |

*Water + HCl (160 °C): water with a pH adjusted to that of CA solution using HCl.

**Supplementary Table S3:** Weight loss percentage of 30% CA solution alone after curing at 140 and 160 °C for 24 h.

| **Samples** | **Weight loss percentage** |
| --- | --- |
| CA-140 | 27±2 |
| CA-160 | 46±3 |


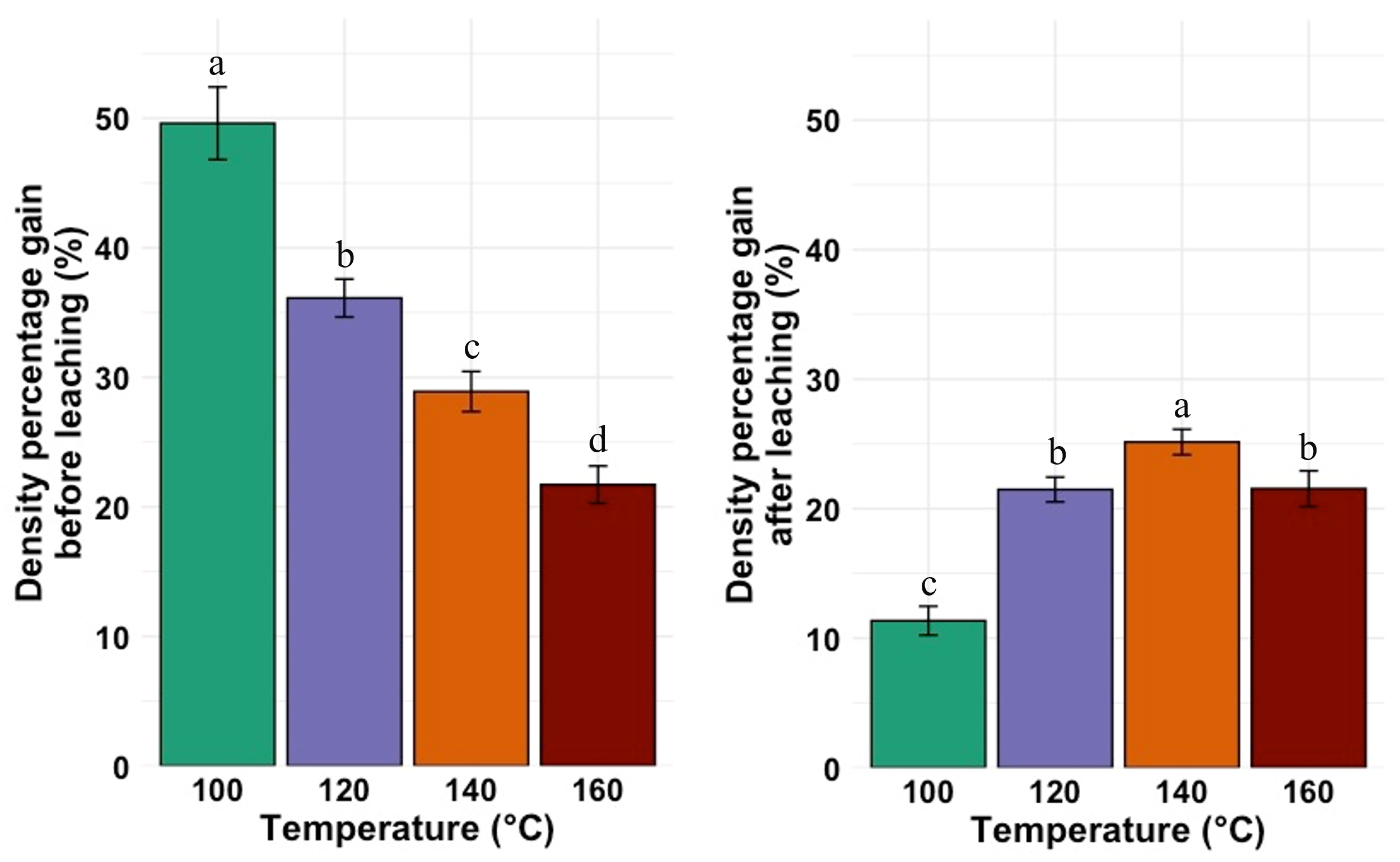


**Supplementary Figure S2:** Comparison of density gain percentage before and after the EN 84 test of wood samples treated with 30% (w/w) aqueous solution of CA at different temperatures. Different superscript letters indicate a significant difference between the curing temperatures at p < 0.05 level.


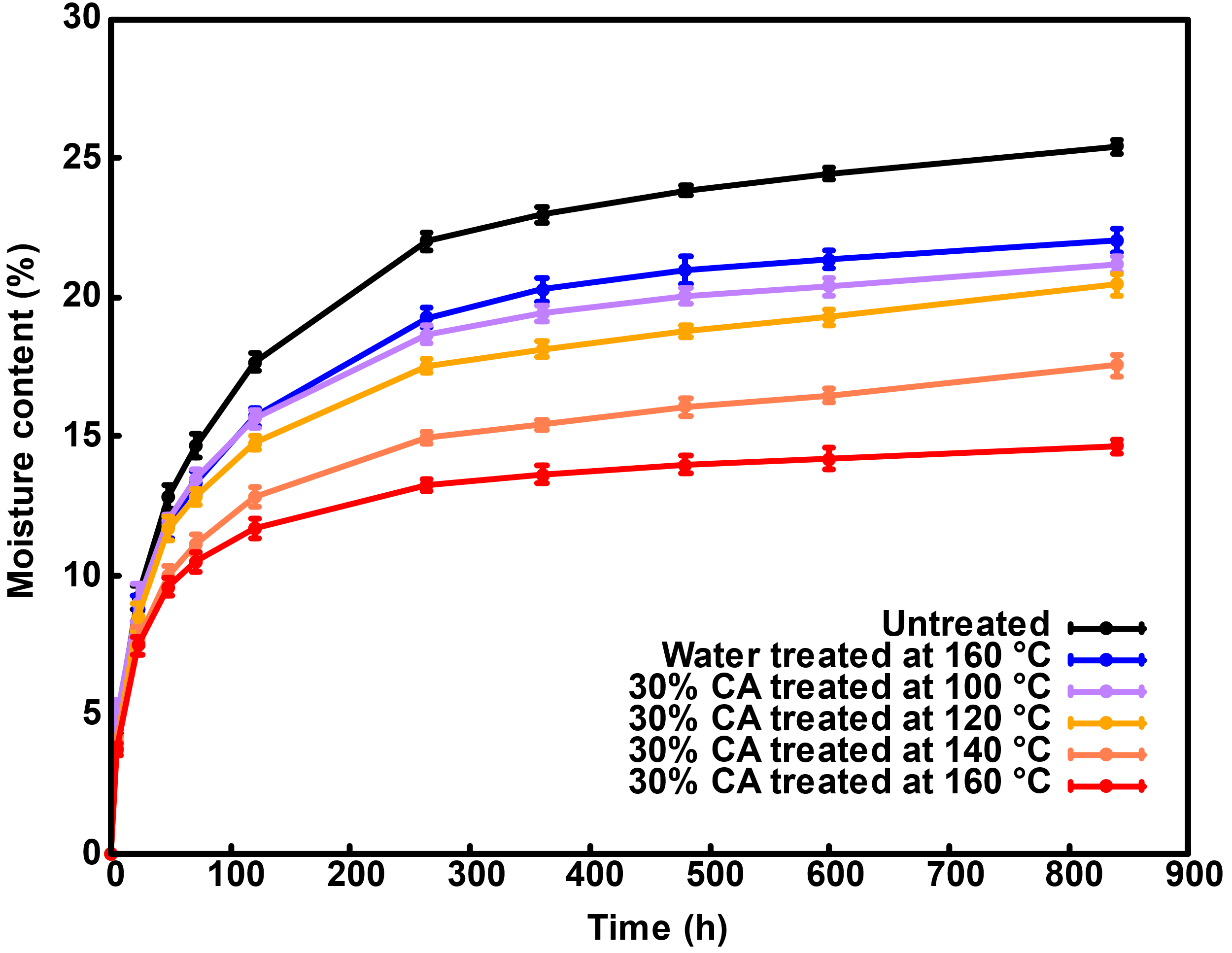


**Supplementary Figure S3:** Comparison of the moisture uptake of untreated, water-treated (160 °C) and 30% CA-treated wood samples at different temperatures.
